# A novel student dataset for ML based effective career growth recommendation

**DOI:** 10.1038/s41598-026-58086-w

**Published:** 2026-06-24

**Authors:** Savitha Acharya, Surendra Shetty, Niranjan N. Prabhu, Nagaraja Shetty

**Affiliations:** 1 Department of Electronics and Communication Engineering, Nitte (Deemed to be University), NMAM Institute of Technology (NMAMIT), Nitte, Karkala, Karnataka India; 2 Department of Electronics and Communication Engineering, Canara Engineering College, Sudhindra Nagar, Benjanapadavu, Mangalore, Karnataka India; 3 Visvesvaraya Technological University, Belagavi, Karnataka India; 4 Department of Master of Computer Applications, Nitte (Deemed to be University, NMAM Institute of Technology (NMAMIT), Nitte, Udupi, 574110 Karnataka India; 5 Department of Aeronautical Engineering, Srinivas Institute of Technology (Affiliated to Visvesvaraya Technological University, Belagavi), Mangalore, 574143 Karnataka India; 6 Manipal Institute of Technology, Manipal Academy of Higher Education, Manipal, India

**Keywords:** Data Mining, Career recommendation systems, Benchmark dataset, Engineering education, Computer science, Electrical and electronic engineering, Engineering, Mathematics and computing

## Abstract

Educational Data Mining (EDM) techniques are increasingly employed to analyze student data for predicting optimal career paths and providing tailored recommendations. A major challenge, however, is the lack of a benchmark dataset that effectively supports this objective, along with the difficulty of identifying the most relevant student attributes for career growth decision support. This study addresses the need for a comprehensive and well-structured dataset to facilitate research on personalized career growth recommendations for engineering students. It presents the methodology used to curate and preprocess a novel benchmark dataset encompassing student demographics, academic background, technical and soft skills, and stress-related factors. Challenges such as data heterogeneity, sparsity, and noise were managed through rigorous data cleaning, feature engineering, and dimensionality reduction techniques.

## Introduction

The rapidly evolving technological landscape demands that engineering education effectively prepare students for successful careers. Effective career guidance and personalized support are crucial for bridging the gap between academia and industry, enabling students to make informed decisions about their future^1^. While traditional career counselling and mentoring are valuable, the Journal of Career Assessment suggests their scalability and ability to personalize guidance for diverse student populations are limited, a gap often filled by the increased accessibility and tailored support offered by online career counselling^2^. The availability of vast amounts of student data, encompassing academic performance, extracurricular activities, skills, interests, and even career aspirations, presents a unique opportunity to revolutionize career support through data-driven decision making^3^. While existing research has explored various aspects of mentoring and recommendation systems, a significant challenge lies in the limited availability of comprehensive and well-structured datasets that accurately capture the diverse needs and characteristics of engineering students.

Research consistently demonstrates the profound impact of mentorship on career development, including increased job satisfaction, higher earning potential, and enhanced career progression^4^. Moreover, personalized recommendation systems can provide crucial guidance by suggesting tailored career paths, identifying relevant skills gaps, and connecting students with appropriate resources and opportunities^5^. By leveraging the power of mentorship and intelligent recommendation systems, we can empower engineering students to make informed career choices, build strong professional networks, and ultimately achieve their full potential.

Machine learning and deep learning offers a powerful approach to personalized career guidance by uncovering complex relationships within student data that traditional methods often miss^6,7^. Specifically, ML algorithms can identify patterns and predict career trajectories, enabling the development of tailored recommendations that empower engineering students to make informed decisions about their future. Some recent research has been done on the use of AI-based educational data mining for predicting students’ dropouts, learning analytics with sentiment awareness, and multimodal educational AI systems through the application of machine learning and deep learning algorithms^8–11^. It is clear that student data becomes more important in creating intelligent education systems.

This paper focuses on the critical first step in this endeavour: the creation of a preprocessed, customized benchmark dataset intended to serve as a basis for future models that generate personalized career growth recommendations for engineering students. Building such a dataset presents significant challenges, including data heterogeneity, sparsity, and the need to capture nuanced aspects of student profiles relevant to career success. This paper details the methodology employed to curate and preprocess this dataset, addressing issues of data cleaning, feature engineering, and data representation. We discuss the rationale behind our data selection and preprocessing choices, emphasizing the importance of creating a robust and representative dataset that is intended to serve as a foundation for developing effective recommendation systems. The resulting dataset, curated in accordance with institutional ethical guidelines and containing sensitive student information, cannot be made publicly available. However, it is intended to facilitate research in personalized career guidance and contribute to the development of tools that support engineering students in achieving their potential. Overall, this work lays the groundwork for future research on recommendation systems and provides a valuable resource for the broader educational community interested in leveraging data to enhance student career development.

**Major Contributions of the study are**:

- Developed a multidimensional benchmark dataset for engineering student career recommendation research.
- Combined multidimensional parameters related to academic, technical, projects, internship, stress-related, and NLP-based features into a single dataset.
- Applied preprocessing techniques to address missing values, noise, sparsity, and dimensionality issues.
- Provided a foundation for future ML-based mentoring and recommendation systems.

## Literature survey

Data cleaning involves identifying and removing inaccurate or irrelevant data. Common data inconsistencies addressed during this process include missing values, outliers, high dimensionality and inconsistent data entries^12^.

### Handling missing data

Missing data is a prevalent issue in career-related datasets, where information about skills, experience, or career aspirations might be incomplete. Missing values in building educational data are typically handled in one of two ways. If the missing data constitutes a small portion of the overall dataset, the affected data samples can be removed, as many data mining algorithms struggle with incomplete data. However, when missing values are more prevalent, imputation techniques can be used to replace the missing entries with estimated values^13^. Missing values have been successfully handled using imputation methods in ^14^, wherein, a small dataset size has been analyzed for prediction of student performance^15^, to predict student dropout and^16^ to predict academic performance of undergraduate students.

### Feature engineering and dimensionality reduction

High-dimensional data, characterized by numerous features can pose computational challenges and negatively impact model performance. Luza et al.^17^ employed feature engineering (FE) techniques, such as redundancy elimination, significance analysis, handling of missing values, and the creation of new features to reduce the dimensionality of academic data of university students in Peru while preserving important variance. Their work demonstrated FE significantly improves the performance of the models, especially in Random Forest (RF) and Logistic Regression (LR), increasing the recall and the AUC ROC (0.94 RF and 0.93 LR).

A wrapper-based forward feature selection method, employing the C4.5 algorithm (J48 classifier), was used to identify the most relevant features from the dataset in^18^. The selection process was validated using 10-fold cross-validation with logistic regression at a 95% confidence level, ensuring only statistically significant features were retained for subsequent modeling. Another study^19^ also used the Forward Selection method to predict the student academic achievement using the Naïve Bayes algorithm yielding an accuracy of 94.43% and an AUC of 0.921 by selecting only 03 of the 33 features.

Uddin et al.^20^ proposed a novel approach to enhance the Feature Engineering and Selection (eFES) Optimization process in ML to identify the optimum set (i.e., grouping) of the right feature set with the finest matching of the feature’s value, leading to optimization of the participating feature, so the ML process can evolve into deciding which features to accept or reject for improved generalization of the model.

To explore ways to make engineering education more sustainable, Poudyal et al.^21^ and Wen et al.^22^ used dimension reduction techniques Principal Component Analysis and Linear Discriminant Analysis to extract key features from their data. The work shows that the dimensional reduction algorithm, followed by the prediction algorithm, achieved the acceptable prediction accuracy for determining student academic performance and career guidance.

### Handling noisy data

Standardization ensures that all features contribute equally to the model and prevents features with larger values from dominating the learning process. To optimize the performance of the chosen machine learning algorithms, pre-college national exam scores (0-100) were standardized (mean = 0, standard deviation = 1)^23^. This preprocessing step addresses potential biases introduced by features with disparate scales.

Recognizing the sensitivity of SVMs to feature scaling, studies employing these techniques often utilize feature scaling as a preprocessing step^14,24^, while. k-Nearest Neighbors (k-NN) is a distance-based method, so feature scaling is crucial^15,16^.

Information gain, a measure derived from entropy that quantifies the reduction in data impurity achieved by feature splits, was employed for feature selection in^25^ to enhance data homogeneity and minimize noise.

Student data can also be noisy^26^, containing irrelevant or inaccurate information. To address potential outliers in student test scores,^27^ employed the Interquartile Range (IQR) method, a robust technique where values falling outside 1.5 times the IQR below the 25th percentile or above the 75th percentile were identified as outliers and Winsorizing was employed to mitigate the influence of these outliers while preserving potentially valuable information, a strategy commonly used to address extreme scores in educational research.

These studies highlight the crucial role of data preprocessing in building effective career growth recommendation systems. The choice of appropriate preprocessing techniques depends on the specific characteristics of the data and the goals of the recommendation system. By carefully addressing issues such as missing values, sparsity, high dimensionality, and noise, researchers can develop more accurate, reliable, and personalized career guidance tools. Table 1 summarizes the existing studies related to student performance analysis and career prediction in terms of dataset attributes, feature extraction techniques, ML algorithms and evaluation metrics.

### Educational datasets

The effectiveness of the machine learning algorithms relies mainly on the educational datasets. These datasets bring together different sources of data that include metrics regarding academic performance such as CGPA, grades, marks, course performance, evaluations of technical and soft skills, coding proficiency, and communication ratings, experience data such as internships, project participation, and co-curricular activities, psychometric tests showing personality traits, aptitude scores, and interests, as well as demographic and socioeconomic attributes^28–30^.

These datasets combine categorical, numerical, and textual features within multi-modal frameworks^31^. Numerical features include academic performance metrics such as CGPA, subject grades, categorical features encompass structural attributes such as company size, organization type, job roles, academic disciplines, professional certifications and psychometric assessments while textual features are extracted from resumes, job descriptions, and narrative skill assessments. These unstructured text sources capture domain-specific language, technical terminology, and implicit skill indicators that numerical and categorical features alone cannot represent. Multimodal integration of behavioral, academic as well as skill-based data has proven particularly valuable in constructing comprehensive student profiles capturing cognitive strengths, skill competencies, behavioral traits aligned with specific career domains^32^.

Furthermore, the literature highlights a significant gap, while extensive efforts have been made to predict academic performance and dropout risk, far fewer studies have been conducted on collecting and analyzing real-time multidimensional student data to create comprehensive career-oriented recommendation systems^33–35^. Recent studies have also emphasized the importance of factors such as fairness, transparency, and interpretability in educational machine learning models, indicating the necessity of a diverse training dataset that is not prone to bias^36^. In this regard, having comprehensive and representative benchmark datasets is essential for developing and testing responsible educational AI systems. Table 2 shows a comparison of existing educational datasets according to feature diversity, recommendation objective, and dataset characteristics, showing that prior work has not achieved much multidimensionality in terms of datasets, which explains why the current benchmark dataset with features from academic, technical, behavioral, stress, and free-text categories has been proposed.

**Table 1:** Summary of existing studies related to student performance analysis and career prediction.

Author	Dataset	Attributes	Feature Extraction Technique	ML Technique	Best Accuracy (%)
Luza et al.^17^	University of Peru	Tutoring, class attendance and academic resources	Redundancy elimination, significance analysis, handling of missing values, and the creation of new features	Random Forests (RF), Logistic Regression (LR)	AUC ROC (0.94 RF and 0.93 LR)
Sumitra et al.^18^	University data 1254 records	Student grades	Wrapper-based forward feature selection method	LR, NB, DT, RF, k-NN, SVM, and MLPNN	95%
Saifudin et al.^19^	UCI dataset	Academic and Demographic	Forward Selection	Naïve Bayes algorithm	Accuracy of 94.43% and an AUC of 0.921
Poudyal et al.^21^	OULAD dataset	Academic and learning behavior information	Principal Component Analysis and Linear Discriminant Analysis	k-NN, DT, and LR	99%
Tatar et al.^23^	357 students CCSIT at IAU	Academic and scores in national exams	Standardization	LR, RF and NB	94.9%
Costa et al.^24^	423 students Brazilian Public University	Academic and demographic	Information gain algorithm	SVM	F-measure − 0.92
Abu et al.^14^	50 students	Academic	MLP-ANN and LDA	SVM	76.3%
Ahmed et al.^16^	800 students Northwestern University	Academic and extracurricular	Genetic algorithms, gain ratio, information gain	K-Nearest Neighbor	91.37%
Iam et al.^15^	811 records Thai University	Academic, demographic, enrolment record	PCA, KPCA	KNN	
Kamal et al.^25^	500 records Panjab University	Academic, demographic, social and behavioural attributes	Information Gain	SVM	98.5%
Majjate et al. ^37^	500 graduates	Demographic, academic, desired University,	Translation, cleansing, and preprocessing	Huber Regressor	MSE = 0.0017, RMSE = 0.0422, R^2^ = 0.9306.
Guleria et al. ^38^	215 instances	Demographic, academic, placement related		Naive Bayes	Recall = 91.2%, F-Measure = 90.7%
Siddique et al.^39^	1227 records	Demographic, academic, family, going out with friends, study time, travel time		MultiBoost with MLP	Accuracy = 98.7%, precision = 98.6%,

Representative educational datasets related to student performance prediction, placement prediction, and career guidance were selected from recent literature for comparison.

**Table 2:** Comparative analysis of existing educational datasets and the proposed benchmark dataset based on feature diversity and recommendation focus.

Reference and Author	Academic Features	Technical Skills	Soft Skills / Behavioral Features	Stress / Mental Health Indicators	Unstructured Text Features	Career Recommendation / Mentoring Focus	Dataset Type
Bhujade et al.^40^	✓	✓	Limited	✗	✗	Placement prediction	Institutional
Nghia et al.^41^	✓	✗	Limited	✗	✗	Dropout prediction	Public (Zenodo)
Raja et al.^32^	✗	✗	✓	✓	✗	Career decision-making	Kaggle
Verdia et al.^33^	✓	✓	Limited	✗	✗	Placement prediction	Not specified
Nizar et al.^42^	✗	✓	✓	✗	✗	Skill assessment	Customized dataset
Menaka et al. ^43^	✓	✓	✓	✗	Limited	Personalized career guidance	LinkedIn & Glassdoor
Syed et al.^44^	✓	✓	✓	✗	✗	Placement prediction	Institutional
Yaqoob et al.^45^	✓	✓	✓	✗	✗	Student performance prediction	Institutional
Villar et al.^46^	✓	✗	Limited	✗	✗	Academic success prediction	Public HEI dataset
Hamoud et al.^47^	✓	✗	✓	Limited	✗	Student performance prediction	Institutional
Proposed Dataset	✓	✓	✓	✓	✓	Career-growth recommendation and mentoring support	Multidimensional benchmark dataset

With the advent of data modeling and feature learning, it is evident that using models like Gaussian Mixture TimeVAE model, Graph Transformer network approach, and adaptive graph neural networks may prove to be advantageous in intelligent feature representation and decision making^48–50^. Using the aforementioned frameworks may facilitate better contextual feature learning and intelligent feature representation through the inclusion of various data types. These methods may be explored further for future research work regarding student profiling and personalized career advice.

## Research gap

The literature encompasses studies predicting various aspects of student success, including academic performance, dropout risk, post-graduation placement status, and appropriate career path selection. Most of these studies demonstrate the effectiveness of machine learning and deep learning techniques in predictive modelling tasks, rather than holistic career-growth recommendation and mentoring systems. Consequently, limited attention has been given in developing comprehensive benchmark datasets that integrate academic, behavioural, and professional attributes within a unified framework.

Despite the growing interest in leveraging data-driven approaches to support student career development, existing datasets in this domain exhibit several critical limitations. Many datasets are narrowly scoped, focusing on specific aspects of student development such as academic performance, while neglecting crucial factors such as soft skills, extracurricular activities, and career aspirations. Moreover, these datasets often lack diversity and sufficient preprocessing, that leads to biased models, missing values, noisy data and limited generalizability. This emphasizes the need for a comprehensive and well pre-processed benchmark dataset to support future research in personalized career-growth recommendation and mentoring systems.

The dataset developed in this study addresses these limitations by combining academic performance, technical and soft-skill proficiency, stress-related indicators, project and internship descriptions, within one unified framework. Compared to most current educational datasets, the proposed dataset offers a wider, multidimensional view designed to support career growth recommendations and mentoring research.

The dataset can help develop and assess machine learning models for analyzing career interests, identifying skill gaps, and offering personalized educational support. Furthermore, the dataset gives a basis for comparing different algorithmic methods and exploring recommendation-focused learning approaches for engineering students.

The study aims to answer the following research questions based on the identified research gaps:• RQ1 : How can heterogeneous student data with academic, technical, behavioural, and soft-skill related attributes be effectively collected and preprocessed into a benchmark dataset for career-growth recommendation research?

- RQ2: What are the appropriate pre-processing and feature-engineering techniques to build a robust educational benchmark dataset for mentoring and recommendation-system applications?
- RQ3: How can we organize multidimensional student-development indicators in a unified framework to facilitate future educational data mining and career-guidance research?

## Methods and materials

### Data collection process

Data collection is a crucial step in the development of any machine-learning model. This study employed a questionnaire-based approach to collect data from students, through Google Forms distributed through scholarly groups on social media. Participants were informed about the purpose of the study and assured of the anonymity and confidentiality of their responses. The collection of these responses was approved by the Research Committee of the Department of Electronics and Communication Engineering, Canara Engineering College, Mangalore, Karnataka, India, and all methods were carried out in accordance with research guidelines and regulations. The questionnaire was designed to gather comprehensive information regarding students’ demographics, academic background, technical skills, soft skills, and stress-related factors. The study considered the experiences of around 200 UG engineering students from various disciplines.

Our participants came from diverse backgrounds and disciplines, with approximately 30% of the sample representing each discipline and a balanced gender ratio of 50% male to 50% female.

**Table 3:** Distribution and characteristics of the sample population used in this study.

Branch	Female		Male		
	Count	Percentage	Count	Percentage	Total
ECE	33	17	35	18.5	68
AIML	30	16	30	16	60
CSBS	35	18.5	26	14	61

### Questionnaire design and features

The questionnaire was structured to capture a range of relevant features, including:

- **Demographics**: Age, Gender, and Location were collected to understand the distribution of the sample population.
- **Academic Information**: Details regarding the student’s Current Education (e.g., degree program, year of study) and Overall CGPA were collected to assess their academic standing.
- **Technical Skills**: Students were queried about their proficiency in various technical skills, including Python, Java, SQL, Data Structures and Algorithms, and Machine Learning. This data was collected to gauge their technical capabilities relevant to potential career paths.
- **Soft Skills Assessment**: To evaluate soft skills, a scenario-based question was presented. Students were asked to provide a written response outlining their approach to the given situation. This qualitative data aims to assess their problem-solving, communication, and decision-making skills in a practical context.
- **Stress Assessment**: Stress levels were assessed using a pre-established questionnaire developed in consultation with a counsellor. This ensured the use of a validated instrument to measure stress-related factors. The specific questions included in this section were designed to capture various aspects of academic stress, time management pressure, and related anxieties.

### Ethical considerations

Participation in the study was voluntary, and students were free to withdraw from the study at any point without consequence. Informed consent was obtained from all participants before they filled out the questionnaire. The data collected was stored securely and anonymized to protect the privacy of the participants.

### Data format and preparation

This structured approach to data collection allowed for the gathering of both quantitative and qualitative data relevant to the research objectives. The use of Google Forms streamlined the data collection process and facilitated efficient data management. The inclusion of a counsellor-developed stress assessment questionnaire ensured the use of a reliable instrument for measuring stress-related factors. The scenario-based soft skills assessment provided context-rich information about the students behavioral competencies. This multifaceted data collection approach provides a robust foundation for analyzing the relationships between technical skills, soft skills, stress levels, and academic performance.

### Data preprocessing

**Fig. 1:**
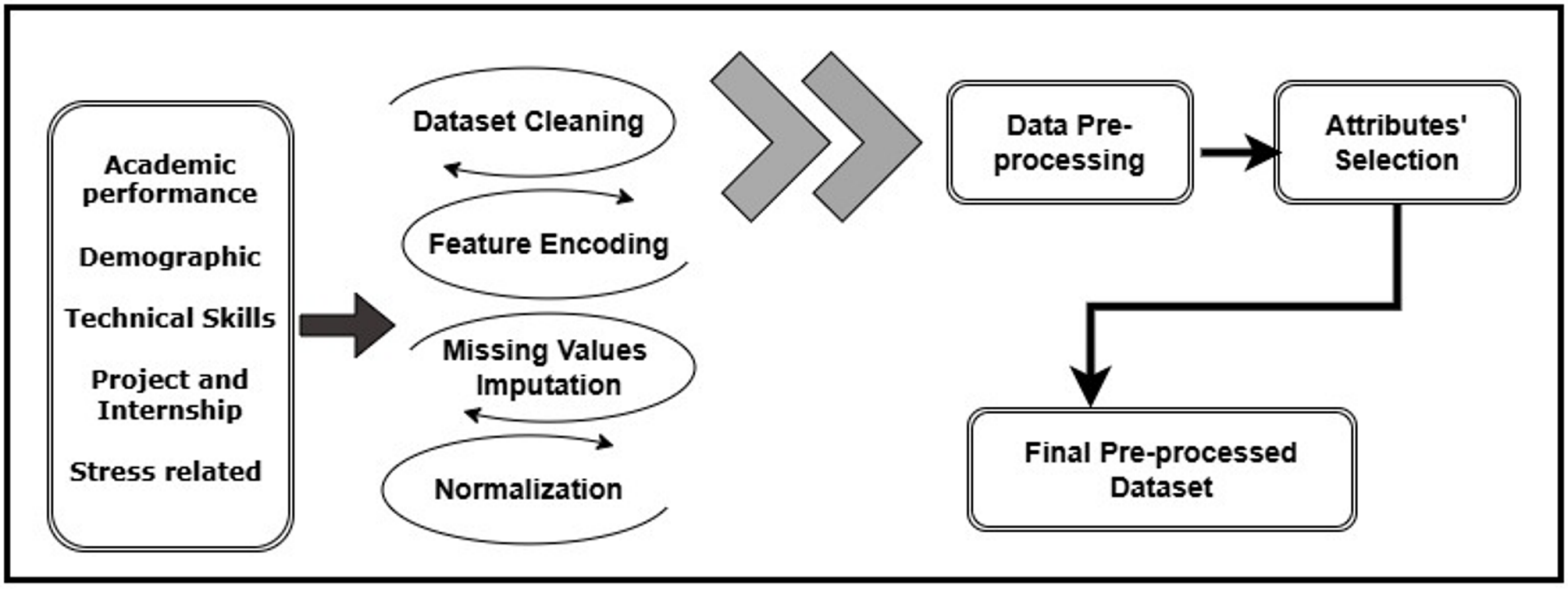
Data preprocessing stages.

Data preprocessing is a crucial step in developing effective career growth recommendation systems. Real-world datasets often contain inconsistencies, errors, and missing values that can negatively impact the performance of machine learning models. Preprocessing techniques such as data cleaning, normalization, and feature engineering are essential to transform raw data into a suitable format for analysis. This process involves handling missing data, removing noise and outliers, and transforming or creating new features to improve the relevance and accuracy of the data. By addressing these issues, we can ensure that the data accurately represents the underlying relationships between student characteristics and career outcomes, leading to more reliable and personalized recommendations. Figure 1. gives an overall picture of the data pre-processing stage.

### Handling missing values

The missing values for the features related to technical skill assessment of students are replaced with the feature mean; this technique is also referred to as “maximum likelihood” ^51^, while the missing values in the categorical variables were imputed by randomly sampling from existing categories within each respective column to preserve the original distribution. For academic data, missing values were imputed using the mean of the student’s marks across all available semesters.

### Clustering methodology for student academic performance and parental background

Clustering^52^ is described as a collection of algorithms used to locate subgroups of observations inside a given data set, which in our case is to identify groups of students with similar academic performance, skill level. We employ clustering, an unsupervised machine learning method that analyzes specific student characteristics within our dataset. This is described by,

$$\:\mathrm{K}={\sum\:}_{m=1}^{M}\:{\sum\:}_{n=1}^{N}{a}_{mn}\parallel\:{i}_{m}-\:{l}_{n}{\parallel\:}^{2}$$

Where M is the total number of data points, N: number of clusters, i_m_: vector of measurement n, l_n_: mean for cluster k, a_mn_: an indicator variable that indicates whether to assign i_m_ to n. We need to determine the value of {a_mn_} and in that gives the least value of K.

This study employed k-means clustering to categorize students based on their academic performance. Prior to clustering, the X and XII percentages were normalized to a 0–10 scale, consistent with the SGPA, to ensure uniform feature scaling. The input features for the clustering algorithm consisted of students’ normalized X percentage, normalized XII percentage, and SGPA scores across multiple semesters, chosen to comprehensively represent their academic trajectory and performance.

To encode the ordinal nature of parental background, we mapped the categorical variables ‘Fath_Qual’, ‘Moth_Qual’, ‘Fath_Emp’, and ‘Moth_Emp’ to numerical values based on predefined rankings of educational qualifications and employment levels. This transformation allowed the incorporation of this ordinal data into subsequent analyses.

K-means clustering was employed to categorize student data in two distinct ways. First, academic performance data was divided into three groups (n_clusters = 3), a number determined using the elbow method. Second, parental background data was clustered into four groups. This dual clustering approach allowed for the identification of key subgroups within the student population, enabling a more nuanced analysis of how academic outcomes relate to differing parental backgrounds.

To optimize convergence and ensure reproducible results, the init = ‘k-means++’ initialization strategy and a fixed random_state = 42 was used. The k-means algorithm iteratively assigns data points to the nearest cluster center and updates the cluster centers based on the mean of the assigned data points. This process continues until the cluster assignments stabilize or a predefined convergence criterion is met. The resulting clusters represent groups of students with similar profiles across two key dimensions: academic performance (based on X percentage, XII percentage, and SGPA scores) and parental background.

### Dimensionality reduction of technical skills and stress related factors

In order to minimize feature sparsity and increase interpretability, a feature engineering and dimensionality reduction procedure was performed for the twenty-two technical skill features. The technical skills were grouped into four broader competency groups according to their functional similarities, curricular relevance, and common association with particular technical domains in engineering education and industrial practice. The four groups included: Programming & Scripting (C, C++, Python, Java, JavaScript, HTML, CSS); Database & Systems (database management, operating systems, cloud computing, and networking); Data Science & Machine Learning (data mining, software development, artificial intelligence, and machine learning); and Embedded Systems & Hardware (embedded C, and HDL).

Stress levels were assessed using a pre-established questionnaire, developed in consultation with a counsellor to ensure a validated measure of stress-related factors, including academic stress and time management challenges. Each question was scored on a 5-point Likert scale and normalized prior to aggregation.

The normalized values were then aggregated using equal weights to generate the overall Stress Index represented by Eq. 1

1 $$SI=\frac{1}{n}\sum_{i=1}^{n} S_i$$

where $\:{S}_{i}$ represents score of the i^th^ question and n denotes the total number of stress indicators.

Equal weighting was intentionally used to preserve simplicity, interpretability, and consistency in terms of representation of all stress parameters in the process of constructing the benchmark dataset. Since this study aims at developing a generalized educational dataset and not building an optimized predictive model, the use of equal weights for each stress parameter enabled the avoidance of any subjective or model-based biases towards certain stress factors. The issue of weighing was also addressed when discussing the questionnaire with the counsellor. Future extensions to this work can explore adaptive or data-driven weighting approaches based on machine learning applications and recommendation system metrics.

The final SI value was linearly scaled to a 0-100 range for interpretability and categorized into four levels: Low (0–25), Moderate (26–50), High (51–75), and Very High (76–100). This unified Stress Index serves as a key feature for subsequent clustering and career recommendation analysis.

### Methodology for categorizing student project and internship activities

**Fig. 2:**
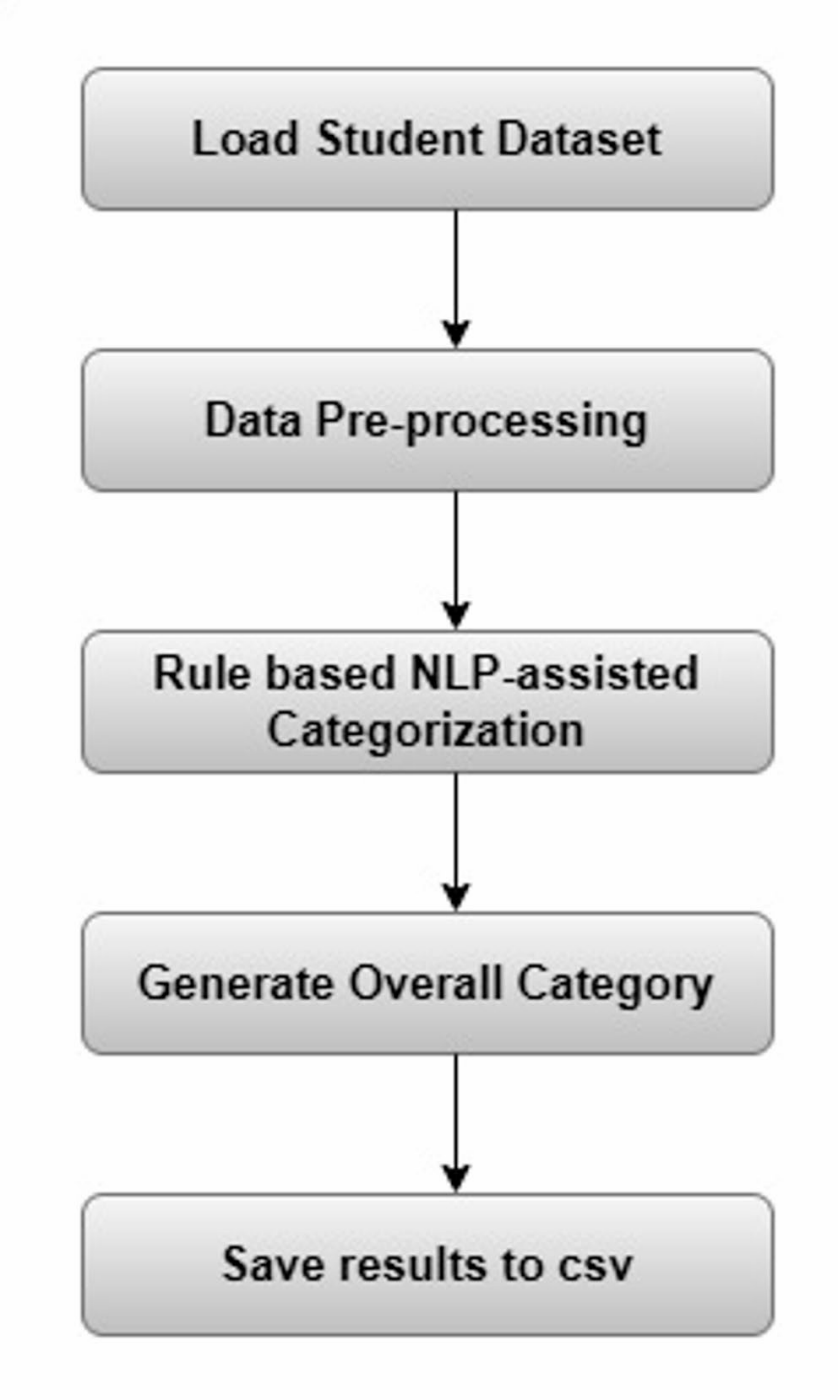
Flow of Rule based NLP-assisted categorization process.

This study employed a two-stage process to categorize student project and internship activities based on their descriptions as in Fig. 2. First, missing values within the Project_Cat and Internship_Cat columns were addressed. Second, a Natural Language Processing (NLP) assisted categorization system was implemented to assign categories to these activities.

To address missing data in the Project_Cat and Internship_Cat columns, we imputed values by randomly selecting from the existing non-missing values within each column. This computationally efficient approach, suitable for categorical data, preserves the data distribution and allows focus on subsequent exploratory NLP-assisted categorization.

We developed a rule-based NLP-assisted categorization approach using spaCy to identify broad project and internship domains such as “Hardware,” “Software,” “Both,” or “Unclear,” based on the presence of predefined keywords. The domain specific keywords were manually curated - Software keywords: {‘python’, ‘java’, ‘AI’, ‘machine learning’, ‘frontend’, ‘API’, ‘web app’}, Hardware keywords: {‘hdl’, ‘Arduino’, ‘Raspberry Pi’, ‘IoT’, ‘microcontroller’}. These keywords were intended to support feature enrichment and consistent categorical representation with the benchmark dataset.

The en_core_web_sm spaCy language model was loaded to provide linguistic analysis of the text descriptions. This approach leverages NLP to identify relevant keywords, offering a more structured categorization than simple string matching. The categorization process was designed as a lightweight and interpretable preprocessing mechanism to assist downstream educational data mining analysis.

The combine_categories function generates an “Overall Category” by prioritizing “Both,” then assigning “Hardware” or “Software” if present in either the project or internship categories. If neither is found, the “Overall Category” is set to “Unclear,” ensuring the combined nature of activities is accurately reflected.

A preliminary internal consistency evaluation was conducted on a small test dataset of 50 samples to assess the coherence of the generated categories which yielded an agreement rate of approximately 88%. A fully labelled ground-truth dataset was not available for this study, which limits the extent of quantitative evaluation; therefore, the internal validation serves as an exploratory test for the rule-based categorization approach rather than a NLP benchmark test. The preliminary evaluation suggests reasonable agreement between the rule-based categorization and manual annotation. However, a larger labelled dataset and comparison with advanced NLP models are required for comprehensive validation. In future, we intend to expand this validation to a larger corpus and compare the results against the existing transformer-based models such as BERT to ensure its robustness.

## Results

### Student academic performance

The K-means clustering algorithm was applied to the academic dataset, comprising six semester SGPA scores, and X and XII percentage scores to explore underlying student-performance groupings. The K-means algorithm was chosen owing to its simplicity in calculations, interpretability, and appropriateness for the exploratory segmentation of multidimensional educational data. Different number of clusters k was tested first based on internal criteria, such as the elbow criterion and visualization, and k = 3 was selected as balance between interpretability and internal clustering metrics. The resulting clustering solution demonstrated a Silhouette Score of 0.288 and a Davies-Bouldin Index of 1.171. The Silhouette Score indicates that the data points are moderately contained inside their assigned clusters with partial overlap among student groups, which is expected in educational datasets. The Davies–Bouldin Index further adds to this, as lower DBI indicates distinct clusters. To validate the effect of preprocessing on these metrics, a comparison of clustering on raw data is done and it is found that the proposed preprocessing pipeline yields approximately 33% relative improvement in the Silhoutte Score along with a reduction in the Davies-Bouldin Index as depicted in the Table 4. However, complete isolation of student groups is unrealistic due to overlapping academic factors.

**Table 4:** Comparison of clustering metrics with and without preprocessing.

Dataset Version	Silhouette Score	Davies–Bouldin Index
Raw Dataset	0.216	1.74
Preprocessed Dataset	0.288	1.171

A more critical interpretation of these metrics indicates that the clustering solution provides exploratory structural trend rather than sharp separated boundaries. This is expected in educational datasets, where student academic performance often overlaps due to shared course structures, common assessment patterns, and similar grading ranges. The Silhouette Score reflects that some cluster boundaries are not sharply defined, while the Davies-Bouldin Index suggests the presence of clusters with comparable internal variation. Together, these measures indicate that although the clusters are not strongly isolated, they are intended to identify broad student-performance pattern within the benchmark dataset.

**Fig. 3:**
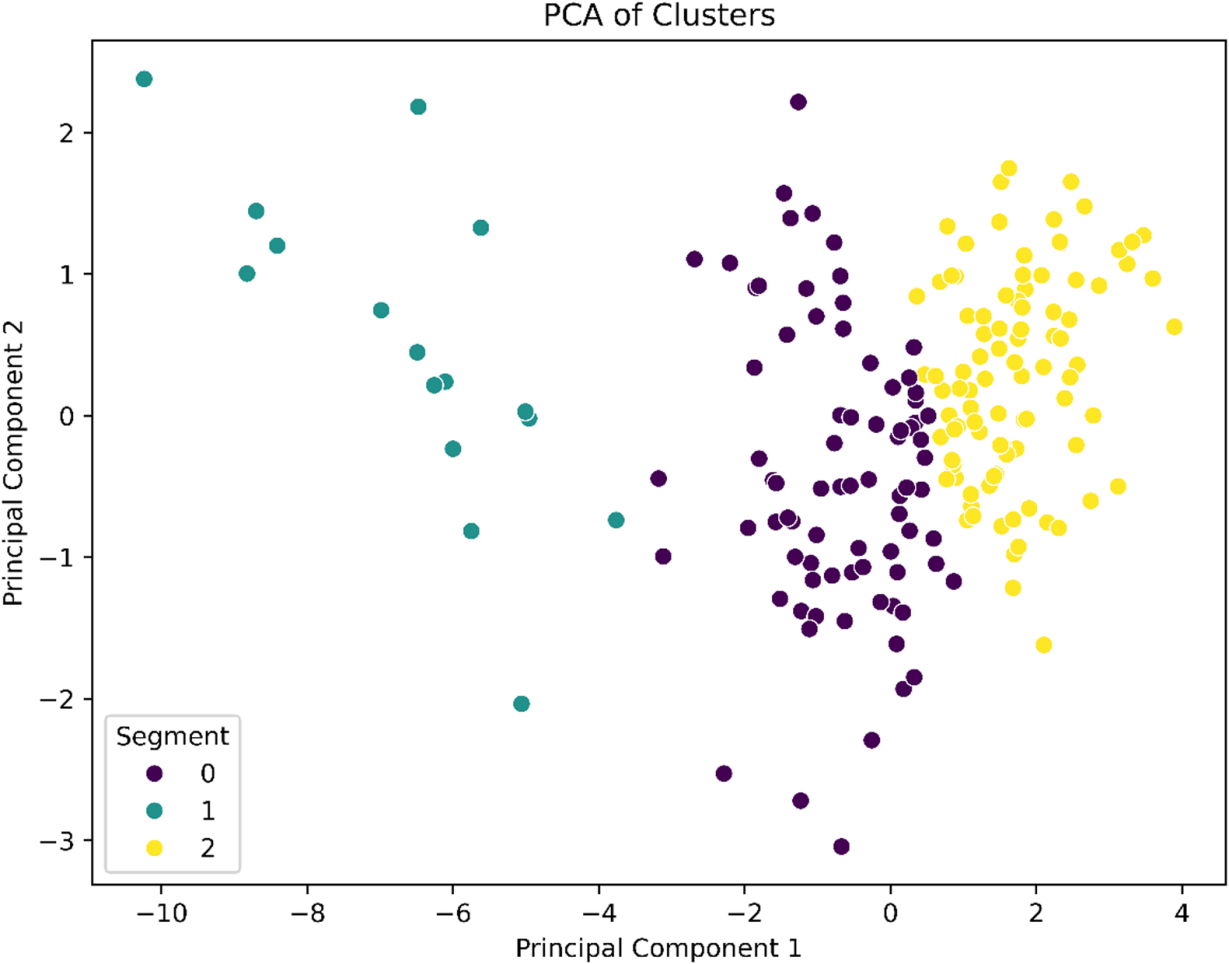
Scatter plot showing the distribution of students academic performance across branches.

To provide a visual representation of the clustering, Principal Component Analysis (PCA) was employed to reduce the dimensionality of the data to two principal components. The resulting two-dimensional scatter plot (Fig. 3) illustrates the separation achieved between the three segments. As shown in the Figure, Segment 2 (yellow) is relatively well-clustered toward higher values of Principal Component 1 (approximately 1.5 to 4.0), reflecting stronger overall performance. Segment 0 (purple) forms a moderately cohesive group centered near 0 to -2 on Principal Component 1, indicating average performance. In contrast, Segment 1 (teal) exhibits substantial dispersion, ranging roughly from − 10 to -4 along Principal Component 1, suggesting higher variability and lower overall performance among these students. The spread and positioning of these clusters align with the underlying academic and skill-based attributes used to form them, offering a clear visual interpretation of the cluster structure.

### Parental background

The distribution of students across parental background clusters provides valuable insights for analyzing student behavior and tailoring career guidance. The clusters are defined as:

- Cluster 0: ‘Good Education & Business’.
- Cluster 1: ‘Highly Educated & Employed’.
- Cluster 2: ‘Poor’.
- Cluster 3: ‘Moderate Education & Financially Good’.

**Fig. 4:**
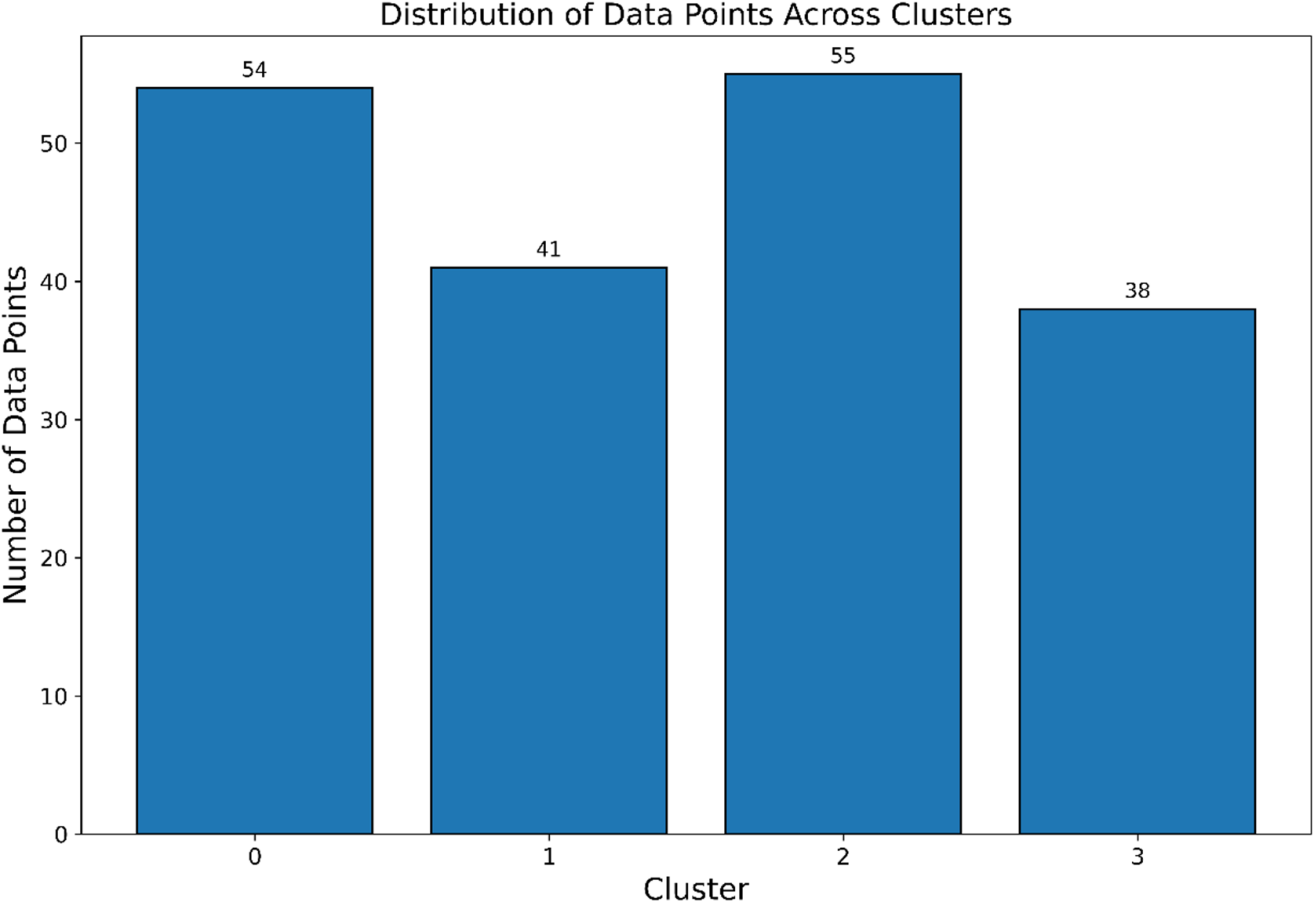
Cluster distribution of parental background based on income ranges and socio-economic groups.

The plot in Fig. 4. reveals that Clusters 0 and 2 contain the largest number of students with approximately 54 and 55 students respectively, while Clusters 1 and 3 include around 41 and 38 students. This distribution indicates variability in the parental-background categories represented in the dataset. Such variation may be linked to differing levels of parental education or socioeconomic stability, which can influence student motivation, availability of resources, and long-term career planning.

Students belonging to Clusters 0 and 1 - typically associated with relatively stronger parental educational or economic backgrounds - may demonstrate higher academic engagement and clearer career aspirations. In contrast, students in Cluster 2, potentially reflecting comparatively lower stability, may require additional academic or psychological support to overcome challenges related to confidence and exposure. Based on this cluster distribution, career guidance strategies can be tailored more effectively: students in Clusters 0 and 1 may be encouraged to pursue advanced degrees and leadership-oriented roles, those in Cluster 2 may benefit from structured mentoring and career stabilization pathways, and Cluster 3 may require a balanced mix of academic reinforcement and career exploration opportunities.

### Student project and internship categories

**Fig. 5:**
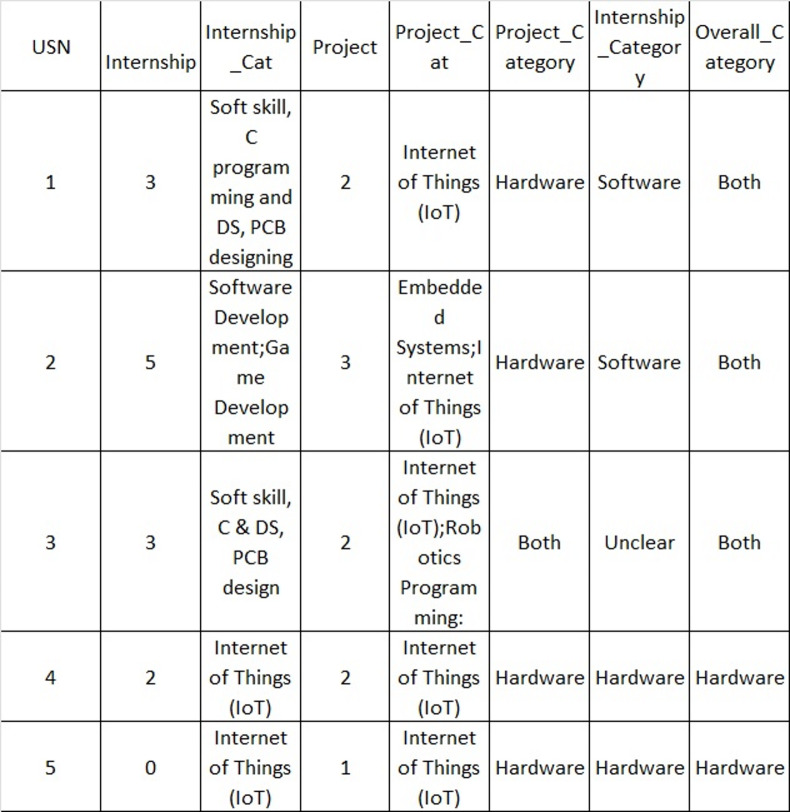
Screenshot illustrating the classification of student project and internship categories using NLP-based keyword grouping.

The combined approach of data imputation and NLP-assisted categorization was applied for grouping the student project and internship categories. This approach, leveraged using spaCy, enabled identification of domain-specific keywords and assignment of categories such as Software, Hardware, Both, and Unclear. As shown in Fig. 5, the resulting dataset includes clearly defined category labels for each student entry, demonstrating the effectiveness of the rule-based NLP pipeline in organizing unstructured text. The distribution in the processed dataset reveals that a substantial portion of students’ projects and internships fall under Software and Hardware, while a smaller number are categorized as Both or Unclear, indicating mixed or ambiguous descriptions. This highlights the variability in student project domains and underscores the value of automated text interpretation for large datasets.

### Data analysis

The dataset was generated by consolidating tables containing academic grades from the Learning Management System (LMS) and students’ responses. Following this, the data underwent translation, cleansing, and preprocessing to ensure consistency before being refined for analysis. Based on this refined student data, a machine-learning model may be trained that may support personalized career recommendations by predicting each student’s fit for various career options. During the data preprocessing step, we analyzed and selected a specific group of features, as outlined in Table 5. The relative importance of these selected features varies in the generation of career recommendations.

**Table 5:** Final set of attributes after preprocessing, feature aggregation, and dimensionality reduction.

Feature	Value	Description
USN	Index	
Gender	0 - Female, 1 - Male	
Branch	0 - ECE, 1 - AIML, 2 - CSBS	
Academic	0 - Average, 1 - Poor, 2 - Good	X, X11, I, II, III, IV, V, VI - columns combined to form one column
Eco_Status	0 - Good Education & Business, 1 - Moderate Education & Financially Good, 2 - Poor, 3 - Highly Educated & Employed	Fath_Qual, Moth_Qual, Fath_Emp, Moth_Emp columns combined to form one column
PGM_SCR	Programming & Scripting – Number	C, C++, Python, Java, JavaScript, HTML, CSS columns combined to form one column
DB_SYS	Database & Systems – Number	DB_MySQL, DB_PostgreSQL, DB_MongoDB, OS_Win, OS_Linux, OS_macOS, Cloud, Network columns combined to form one column
DS_ML	Data Science & Machine Learning – Number	Data Min, Sw Dev, Al, ML columns combined to form one column
EMB_HW	Embedded Systems & Hardware - Number	Emb_c, HDL columns combined to form one column
Pro_Int_Hac_No	Number	Values in Hackathon, Internship and Project columns added to form one column
Pro_Int_Cat	0 - unclear, 1 - hardware, 2 - software, 3 - both	Internship_Cat and Project_Cat columns combined to form one column
Stress	0 - Low, 1 - Moderate, 2 - High, 3 - Very High	Last 20 columns of questionnaire on stress assessment is reduced to one column

### Data visualization

Data visualization^53^ is crucial both for exploring the relationships within our student dataset and for effectively presenting our findings. Visual representations of student data, such as skill distributions and correlations between academic performance and technical interests, allow for a deeper understanding of individual student profiles and the identification of key characteristics relevant to career success.

**Fig. 6:**
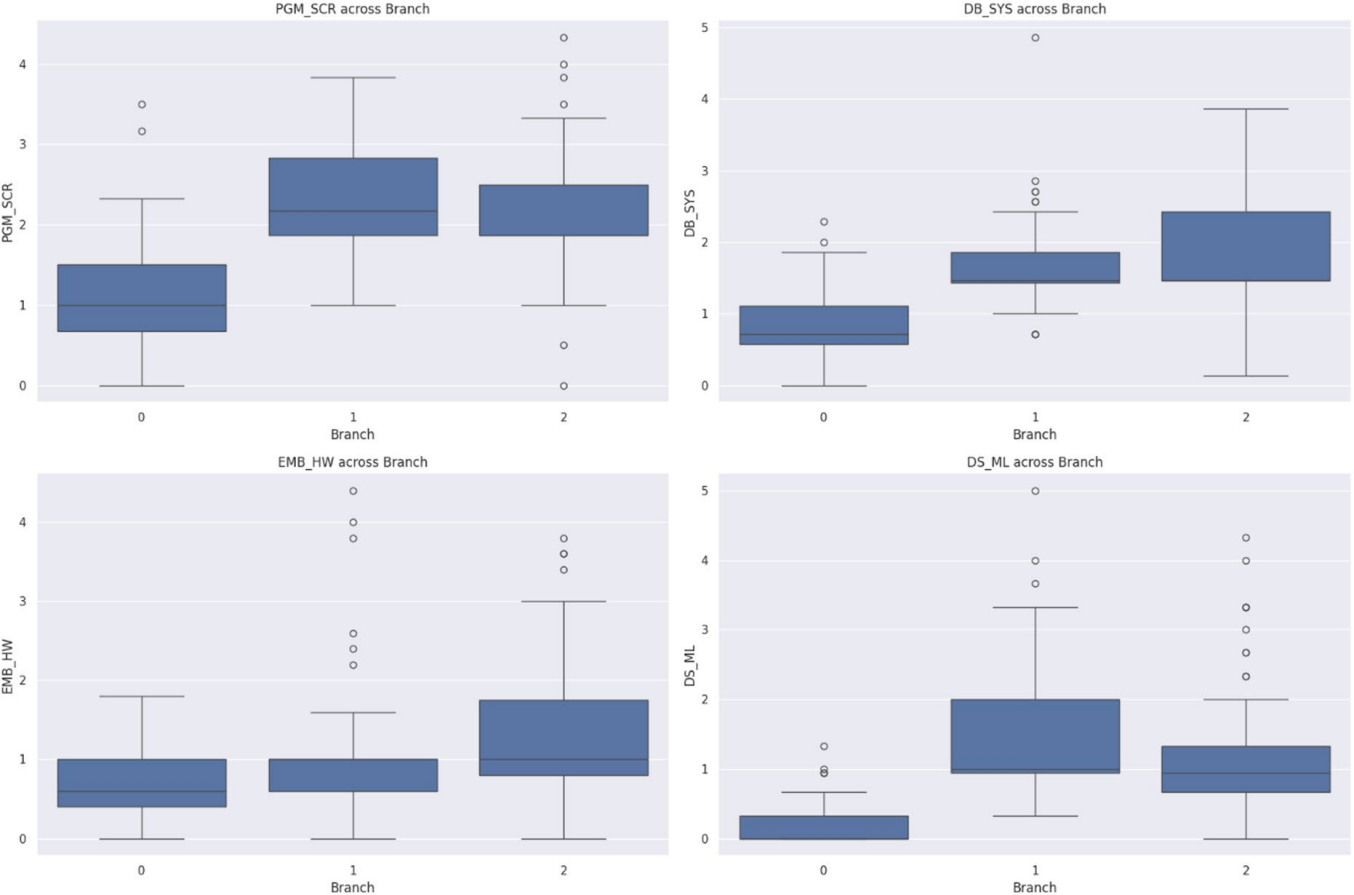
Boxplot of technical skill distributions across different branches for aggregated skill groups.

Figure 6 shows the boxplot of the technical skill distribution across various branches. According to this, Branch 2 consistently exhibits higher median scores in most of the measured areas (PGM_SCR, DB_SYS, DS_ML). This suggests that Branch 2 might represent a higher-performing group or a branch with a specific focus on these subjects. Branch 1 often shows more variability or spread in the scores, indicating a potentially diverse range of performance within that branch. Potential Outliers: The presence of outliers suggests some individuals or data points with unusually high or low scores compared to their group. These could be worth further investigation (e.g., exceptionally talented students or students needing additional support). Additionally, quantitative inspection of the boxplots shows that Branch 2 attains higher median values across PGM_SCR (approximately 2.2), DB_SYS (approximately 2.4), and DS_ML (approximately 1.8) compared to Branch 0 and Branch 1. Branch 1 displays the widest interquartile ranges, confirming greater score dispersion. Across all branches, several upper and lower outliers are visible, highlighting a small subset of students who deviate substantially from the group average. These quantitative differences reinforce the need for branch-specific academic support and targeted skill development strategies.

**Fig. 7:**
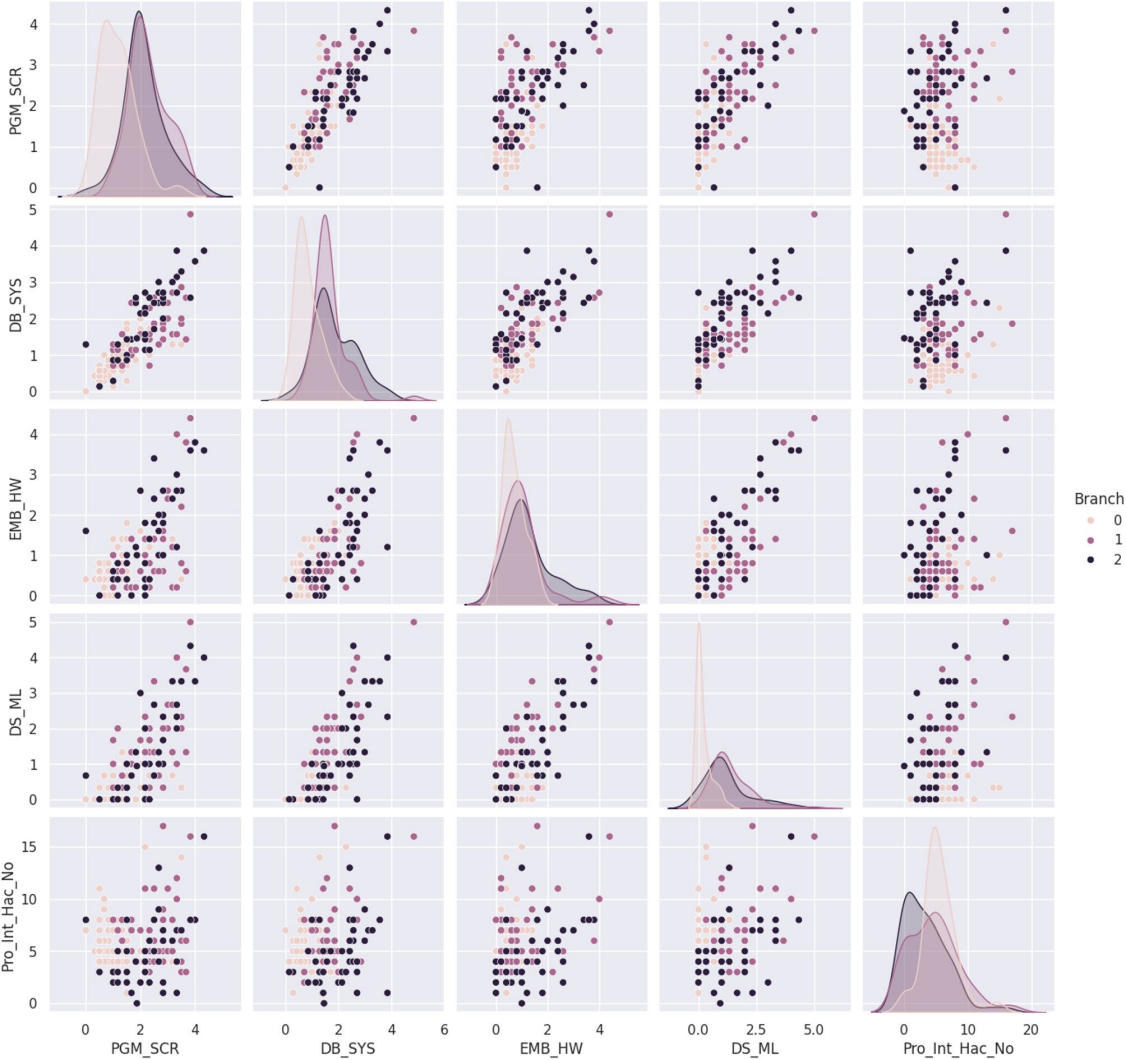
Correlation plot between technical skill levels and participation in technical events.

We used a pair plot^53^, as shown in Fig. 7, to visually represent scatter plots of technical skill attributes and participation-related variables. The pair plot, highlighted distinct patterns, revealing that higher technical skills were associated with higher participation in technical events indicating a moderate positive relationship within the dataset. The analysis further revealed that the pairwise scatter plots show moderate positive correlations between PGM_SCR and DB_SYS (*r* ≈ 0.45), and between DB_SYS and DS_ML (*r* ≈ 0.41).

The density curves also reveal that Branch 2 tends to cluster toward higher skill values, whereas Branch 0 displays lower concentration across most axes. The increasing trend visible in the Pro_Int_Hac_No axis indicates that students with higher technical skills generally engage in more technical events (up to 15–18 events), reinforcing the pattern observed in the boxplots and supporting the overall consistency of relationships within the dataset.

Most variables (PGM_SCR, DB_SYS, EMB_HW, DS_ML) appear to have a roughly normal distribution, although some might be slightly skewed. Furthermore, students belonging to Branch 2 (represented by the yellow/orange color) consistently demonstrate higher scores across different skills compared to other branches.

This pair plot provides meaningful insights between technical skills, participation and branch-specific trends. These insights may support future recommendation systems by highlighting the relevance of technical participation, technical skills, and potential interactions between these factors in generating career recommendations.

### Conceptual framework for future career recommendation applications

The curated dataset developed in this study is intended to support further research on personalized career recommendation and mentoring in engineering education. To illustrate this, a conceptual career recommendation framework is proposed consisting of four main components: (i) feature preprocessing and encoding, (ii) integration of dimensionality-reduced academic, technical, soft-skill, and stress-related indicators, (iii) a machine learning candidate model module, and (iv) an NLP-assisted interest mapping layer. These components together form a hybrid pipeline designed to explore the feasibility of predicting suitable career pathways from the curated dataset.

The framework integrates multidimensional student attributes such as academic, technical, soft-skill, and stress indicators to support the exploratory development of potential career prediction models using supervised or unsupervised learning approaches. Additionally, the incorporation of NLP-based project and internship categories may enable interest-based profiling and exploratory recommendation-focused researches. The proposed preprocessing methodology adopted in this study ensures consistency in the presentation of features, decrease in noise levels, as well as proper incorporation of diverse student attributes, thus making it possible to use this approach as a basis for future model development and experiments. As the primary focus of this study is dataset development, full model training, optimization, and validation are planned for future work. Preliminary evaluations, where applicable, are limited to internal checks to ensure feature coherence and do not represent deployable model performance.

**Fig. 8:**
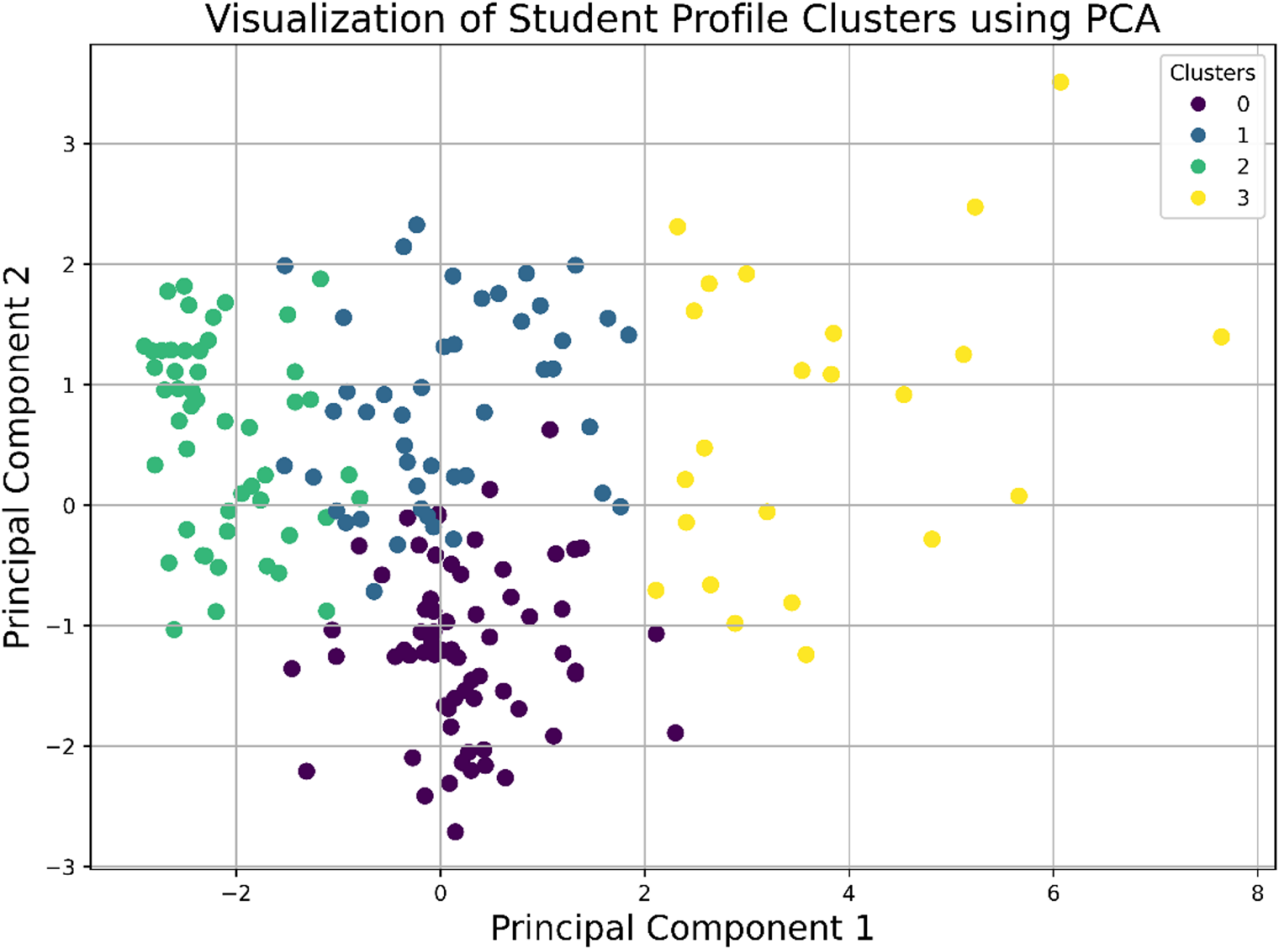
Scatter plot showing the distribution of student profile generated using K-Means clustering on the multidimensional benchmark dataset.

**Fig. 9:**
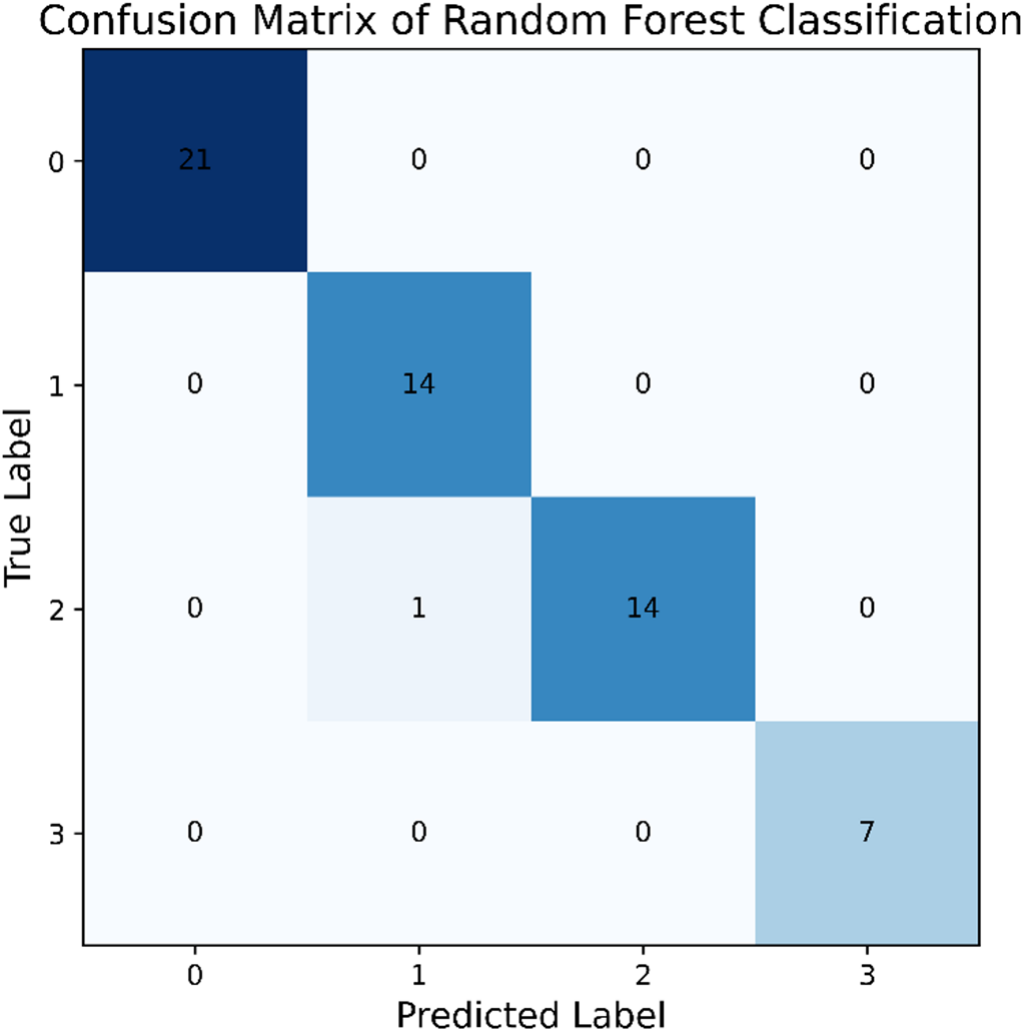
Confusion matrix of the Random Forest classifier trained using the generated dataset.

In order to provide the preliminary baseline evaluation to demonstrate the structural usability of the proposed benchmark dataset, K-Means clustering technique was applied to generate the pseudo labels of multidimensional student profile groups. The K-Means clustering procedure was applied in the normalized feature space with respect to the academic, technical, behavioural and stress-related attributes of the students. Consequently, the obtained cluster labels served as the target class labels for the Random Forest classifier in order to test the ability of the features to form the meaningful patterns. The data set was split into training and test sets with a ratio of 70:30 before classification. The classifier achieved an accuracy of 98%, on the generated pseudo-labels, suggesting that the curated feature space contains distinguishable multidimensional student-profile patterns. This is merely presented as a preliminary consistency result for the feature space and not as an indicator of the accuracy of the career recommendations. Figure 8 shows the distribution of clustered groups of students in the reduced feature space of two dimensions obtained from academic, technical, behavioral and stress factors indicating that there are distinguishable multidimensional patterns existing in the curated educational benchmark dataset. Figure 9 shows the classification performance of the baseline model for clustering multidimensional groups of student profiles obtained from the curated benchmark dataset. The results above show that the feature space is consistent and learnable. The present evaluation is intended as a baseline assessment of dataset usability and feature-space consistency rather than a comprehensive comparison of predictive models. The development and benchmarking of multiple machine-learning algorithms remain part of future work. This preliminary study was not aimed at the construction of the optimal recommendation system, but rather to provide some initial results concerning the usability of the dataset in question.

## Conclusion, limitations and future work

The study addressed the need for a well-organized dataset, designed to support research on personalized career growth recommendations for engineering students. A multidimensional dataset was meticulously curated and preprocessed, encompassing a wide array of student attributes, including demographics, academic history, technical, soft-skill, and stress-related factors. The proposed preprocessing framework addressed challenges, including data heterogeneity, sparsity, and noise, through data cleaning, feature engineering, normalization and dimensionality reduction techniques. By providing this foundation, the work contributes to enhancing the understanding of student career development and supports further research within the educational community.

Despite its contributions, this study has certain limitations. The sample size, though sufficient for exploratory analysis, constrains the statistical robustness, external validity and generalization capability of large-scale predictive models. In addition, the use of data collected from a single institution may introduce sampling bias and limit the diversity of student experiences represented in the dataset. Moreover, the dataset originates from a single institution, which may limit its applicability across diverse student populations, educational settings and geographical regions. The stress assessment relies on self-reported values, introducing potential subjective bias, and the rule-based NLP categorization used for project and internship descriptions may not capture deeper contextual meaning. Furthermore, the clustering analysis was intended primarily for exploratory dataset characterization. Alternative clustering algorithms and cluster stability analyses were not investigated in the present study, which may limit the assessment of clustering robustness.

Future work will therefore focus on expanding the dataset to include a more diverse student population, which will enhance the robustness, validity and cross regional applicability of the findings. Moreover, comparative analysis involving the use of other clustering techniques like hierarchical clustering and DBSCAN could be included in future studies for more insights into clustering validity. Additional efforts will involve validating advanced machine learning models, comparing rule-based NLP methods with transformer-based models such as BERT, integrating socio-economic and psychological variables, and ultimately developing a deployable end-to-end career recommendation system.

In addition, future research could incorporate explainable AI techniques to improve the transparency of career recommendations and explore longitudinal data collection to examine how student skills, interests, and stress levels evolve over time. More advanced architectures-such as graph-based recommenders or multimodal deep learning can also be evaluated using richer ground-truth labels derived from student placement outcomes. These extensions highlight the broader potential of the dataset for developing robust and interpretable career recommendation systems.

## Data Availability

All the data used in the article have been made available in the present article. The data that support the findings of this study are available from the corresponding author, Nagaraja upon reasonable request.
